# An All-Glass Microfluidic Network with Integrated Amorphous Silicon Photosensors for on-Chip Monitoring of Enzymatic Biochemical Assay

**DOI:** 10.3390/bios7040058

**Published:** 2017-12-05

**Authors:** Francesca Costantini, Roald M. Tiggelaar, Riccardo Salvio, Marco Nardecchia, Stefan Schlautmann, Cesare Manetti, Han J. G. E. Gardeniers, Giampiero de Cesare, Domenico Caputo, Augusto Nascetti

**Affiliations:** 1 School of Aerospace Engineering, Sapienza University of Rome, via Salaria n. 851/881, 00138 Rome, Italy; marco.nardecchia@uniroma1.it (M.N.); augusto.nascetti@uniroma1.it (A.N.); 2 Department of Chemistry, Sapienza University of Rome, p.le Aldo Moro n.5, 00185 Rome, Italy; riccardo.salvio@uniroma1.it; 3 Mesoscale Chemical Systems, MESA+ Institute for Nanotechnology, University of Twente, P.O. Box 217, 7500 AE Enschede, The Netherlands; r.m.tiggelaar@utwente.nl (R.M.T.); s.schlautmann@utwente.nl (S.S.); j.g.e.gardeniers@utwente.nl (H.J.G.E.G.); 4 NanoLab cleanroom, MESA+ Institute for Nanotechnology, University of Twente, P.O. Box 217, 7500 AE Enschede, The Netherlands; 5 Department of Environmental Biology, Sapienza University of Rome, p.le Aldo Moro n.5, 00185 Rome Italy; cesare.manetti@uniroma1.it; 6 Department of Information Engineering, Electronics and Telecommunications, Sapienza University of Rome, Via Eudossiana, 18, 00184 Rome, Italy; giampiero.decesare@uniroma1.it (G.d.C.); domenico.caputo@uniroma1.it (D.C.)

**Keywords:** microfluidic channel, surface functionalization, amorphous silicon photosensors, chemiluminescent based assay, lab-on-chip

## Abstract

A lab-on-chip system, integrating an all-glass microfluidics and on-chip optical detection, was developed and tested. The microfluidic network is etched in a glass substrate, which is then sealed with a glass cover by direct bonding. Thin film amorphous silicon photosensors have been fabricated on the sealed microfluidic substrate preventing the contamination of the micro-channels. The microfluidic network is then made accessible by opening inlets and outlets just prior to the use, ensuring the sterility of the device. The entire fabrication process relies on conventional photolithographic microfabrication techniques and is suitable for low-cost mass production of the device. The lab-on-chip system has been tested by implementing a chemiluminescent biochemical reaction. The inner channel walls of the microfluidic network are chemically functionalized with a layer of polymer brushes and horseradish peroxidase is immobilized into the coated channel. The results demonstrate the successful on-chip detection of hydrogen peroxide down to 18 μM by using luminol and 4-iodophenol as enhancer agent.

## 1. Introduction

With the rapid spread of microfluidic systems for biochemical assays, there is an increasing need for highly integrated lab-on-chip systems that enable the combination of different analytical procedures on a single device, ranging from sample preparation to the transduction of the analytical information into an electrical signal suitable for processing purposes. The result is a biosensing-integrated device with applications such as drug discovery, medical diagnostics, food quality control and environmental monitoring [1,2,3,4]. 

The integration of biosensors into microfluidics technology offers a miniaturized platform with several advantages over the conventional laboratory methods. These advantages are: (a) reduction of sample and reagent volumes, improved mixing, higher energy efficiency and reduced analysis time, (b) scalability, safety and lower waste production, leading to cost reduction and superior control of reaction conditions, (c) the possibility to carry out parallel processing; and (d) to perform in-the-field or point-of-care measurements.

Up to now, microfluidic systems have been successfully applied for protein analysis, determination of enzyme activity, immunoassay and genetic analysis [1,5,6,7].

Microfluidic devices can be fabricated from different types of materials [5]. Initially, silicon was largely applied for the fabrication of microfluidic chips, stimulated by technological developments in microelectronics. Nevertheless, in order to integrate several steps of the analytical procedure within these systems, other materials were also applied. Nowadays, glass is frequently used as a substrate because of its favorable properties such as chemical inertness and optical transparency. Moreover, polymers such as poly(methyl methacrylate) (PMMA), polycarbonate (PC) and polydimethylsiloxane (PDMS) [8] are used for the realization of microfluidic devices. Polymers are cheaper than glass for mass-production; however, they present some disadvantages such as higher hydrophobicity, which might lead to problems upon filling microfluidic channels with water solutions. Another drawback is the adsorption of biomolecules that might interfere with analytical protocols. Therefore, polymer microfluidic networks need additional treatments in order to achieve hydrophilic surfaces and reduced non-specific surface adsorption [5]. Recently, paper and thread have also been used for the fabrication of microfluidic chips [4,9].

The step from a purely microfluidic device to a lab-on-chip system requires the integration of different steps of the traditional laboratory procedure into the microfluidic platform. Thus, in order to develop a lab-on-chip device as biosensor for a given target, it is fundamental to devise: (i) a strategy to introduce a small volume of sample into the device, (ii) a method to chemically functionalize the interior of the microchannels (surface functionalization) for integrating biomolecule(s) into the device, (iii) a detection method for monitoring/sensing the target, (iv) a transduction system to convert the detected information into an electrical signal, possibly digital, suitable for processing and (v) a proper technological process that enables to embed sampling, surface treatments and detection into the same platform.

Small sample volumes can be introduced into lab-on-chip systems using pressure-, electrokinetic- or capillary-driven flow. In devices that use electrokinetic-driven flow, sample movements are controlled by applying an electrical potential. Pressure-driven flow is often generated by a syringe pump exerting positive pressure to push the fluid, but negative pressure can also be used. Capillary flow is also widely applied for microchannel loading [1].

Microfluidic systems require precisely tailored surface properties for many analytical applications. Nowadays, a variety of surface modification methods are available for the immobilization of biomolecules acting as biosensors. These methods rely on simple absorption to a support of more sophisticated approaches involving covalent bonding, bioaffinity bonding and sol-gel entrapment [1]. The biological recognition-sensing element dictates the selectivity and specificity that allow the biosensor to respond to a specific target. Antibodies, aptamers and enzymes were immobilized by us into lab-on-chip devices for a variety of bioanalytic applications [2,10,11,12,13,14].

The on-chip integrated detection method must sense the interaction between the sensing element and the target. The detection strategies can be classified as optical [15] or electrochemical [16]. Among the optical detection methods, biosensors relying on fluorescence or absorbance spectroscopy are commonly used in microfluidic applications [17]. However, fluorescence spectroscopy requires the integration of complex optics and sometimes quantitative analysis can be hindered by the auto-fluorescence generated by the polymeric material of which the microfluidic chip is made. Although absorbance is the standard detection method employed in most bioanalytical applications, the relatively short absorbance path lengths available in microfluidic devices (at most a few millimeters) can result in a decreased sensitivity [16]. Chemiluminescence (CL), which is widely used for biochemical assays [18], is an alternative detection method for microfluidics since it does not require an excitation source (and inherently no optical path length) and does not suffer from background signal(s). Moreover, chemiluminescence typically has a very high specificity and sensitivity [16].

Most chemiluminescent biosensors rely on conventional laboratory instrumentation for the transduction of the optical signal into a digital signal. Examples include inverted optical microscope or CCD cameras [16], waveguides and in-line detection elements [19]. However, the use of transducers undermines the advantages of portability and simplicity of the microfluidic device. Therefore, in recent years, some groups started to work on the implementation of optical sensing elements directly on the microfluidic platform (on-chip integration). In detail, photosensors are integrated in/on silicon or PDMS microfluidic chips [20,21,22,23,24] for analytical application. 

In this work, we present an innovative lab-on-chip system based on a glass microfluidic network with on-chip amorphous silicon photosensors (a-Si:H) [25] for the monitoring of chemiluminescent-based enzymatic biochemical assays [26]. The position of the sensors along the microfluidic network enables the precise monitoring of the reaction kinetics. A layer of polymer brushes [10,27,28,29] was grown on the inner wall of the microchannel and subsequently an enzyme was immobilized to it by covalent bonding. As a proof of principle, horseradish peroxidase (HRP) was selected as model enzyme to be anchored for detecting hydrogen peroxide (H_2_O_2_), which plays an important role in atmospheric and biochemical processes [30].

## 2. Materials and Methods

*Fabrication of the glass chips with integrated amorphous silicon photosensors*. In order to obtain a lab-on-a-chip system with combined functionality—i.e., a glass microfluidic network coated with an enzyme for biochemical analysis based on chemiluminescence (CL) and on-chip measurement of this CL-signal with integrated photosensors—the design and fabrication sequence of such system needs to fulfill several requirements. In fact, within the design phase the following two aspects need to be taken into account:

1. the fabrication process of the thin-film amorphous-silicon photosensors should not affect the interior of the microfluidic glass network. More specifically, the chemical surface composition of the glass channels is important for the enzymatic functionalization protocol (which is based on silanol groups), and thus the materials (gases, liquids) involved in the procedure to realize photosensors should not enter the fluidic network.
2. the chemical treatment and functionalization of the microfluidic interior should not affect the functionality and performance of the thin film amorphous silicon photosensors.

**Figure 1:**
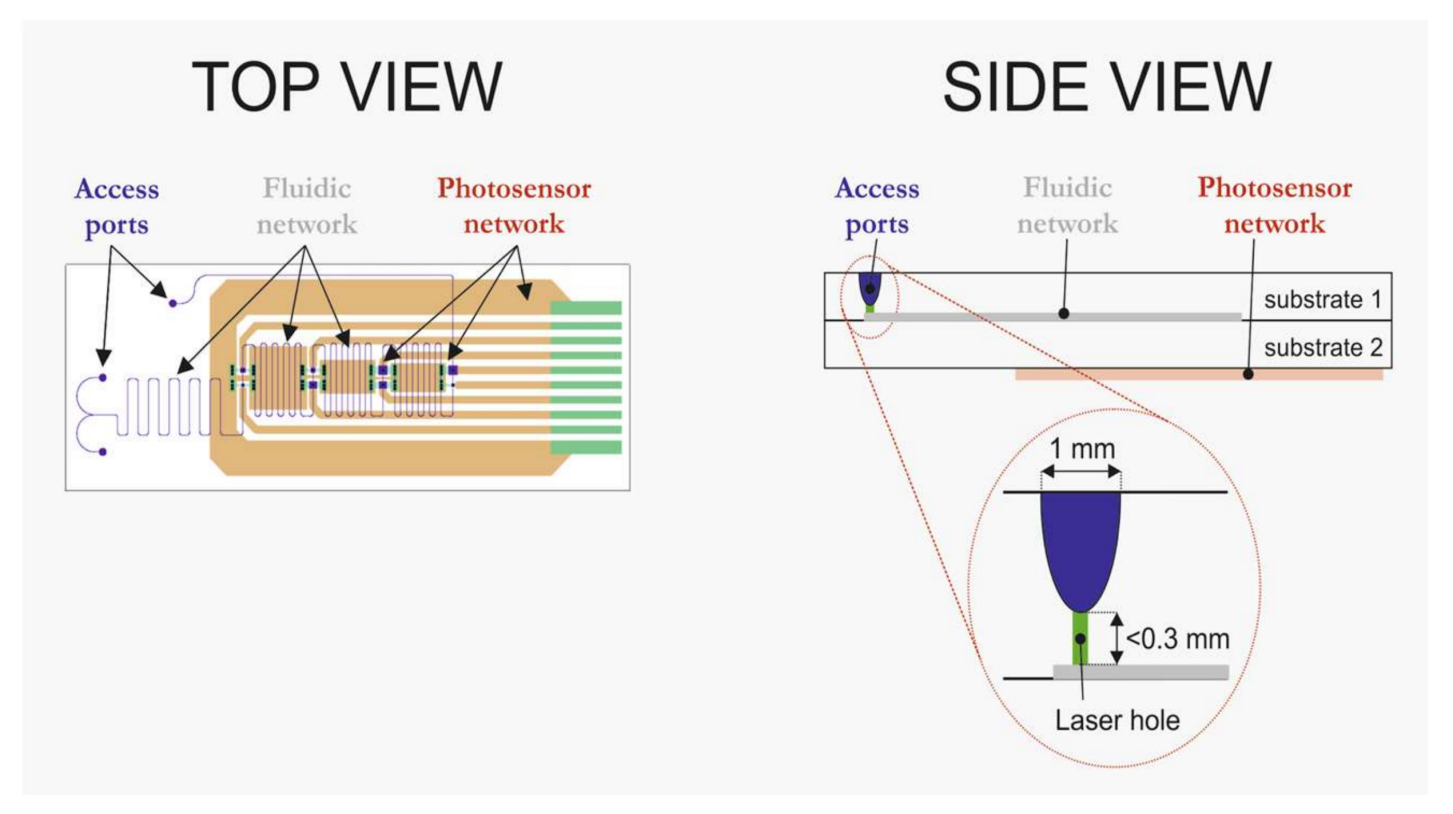
Schematic views of the design of the all-glass chip with integrated photosensors.

In order to fulfill these aspects, the photosensors and the accesses to the microfluidic network are separately located in the chip-design. In the schemes in Figure 1 it can be seen that the access ports are on one side of a chip, whereas the photosensors are located on the opposite side. Electrical readout/control of the sensors are accomplished by means of a slider-connection on the right-hand side, whereas the fluidic connection is realized with a customized holder on the left-hand side of the system.

In Figure 1 it can be seen that the connection between the fluidic access ports and the microfluidic network is formed via a cylindrical-shaped holes made by laser drilling. These laser holes are made after the realization of the fluidic network, glass-glass bonding and deposition of the a-Si:H photosensors. This sequence of steps ensures that no materials (gases, liquids) of the PECVD-based fabrication process of the photosensors will enter the fluidic network, and thus that the interior glass walls are preserved. Thus, the chip fabrication sequence is as follows:

1. realization of fluidic networks on the bondside of a 1.1 mm thick Borofloat 33 (BF33) substrate;
2. realization of powderblasted pits in the 1.1 mm thick BF33 substrate (at the non-bond side). These powderblasted pits are the fluidic access ports (note: these pits are not yet in contact with the microfluidic channel network);
3. realization of thin film amorphous silicon photosensors on the stack. Sensors are deposited onto the 0.5 mm thick substrate, so that the sensors are as close as possible to the fluidic network;
4. realization of fluidic accesses to the microfluidic network, by means of laser drilling at the bottom of the predefined powderblasted pits;
5. realization of individual chips via dicing of the BF33 stack.

Fabrication of 40 mm long, 15 mm wide and 1.6 mm thick BF33 chips with a layout as shown at the left-side of Figure 1 is done in 100 mm diameter BF33 substrates. Each chip comprises two inlets and one outlet. Fabrication of the serpentine-shaped fluidic network and 0.9 mm deep powderblasted pits in 1.1 mm thick substrates, as well as bonding of these substrates to 0.5 mm thick BF33 substrates, is done by following the fabrication procedure previously reported [31]. In short, the fluidic network is isotropically etched in BF33 using 25% hydrofluoric acid (HF) and 10 µm wide lines as mask-pattern, yielding a total channel length of ca. 240 mm with a (nearly) half-circular cross-section (depth of 50 µm; (top) width 110 µm). Alignment of the powderblasted access pits to the fluidic structure, as well as of the a-Si:H photosensors, is performed by means of alignment marks that are defined into the glass upon realizing the microfluidic network. After a channel length of 50 mm, a set of two a-Si:H sensors is positioned on the channel, and sets of two sensors are also located on the channel after a length of 100, 150 and 200 mm respectively (*sensor area are: after 50 mm 0.04 and 0.09 mm^2^, after 100 mm 0.09 and 0.25 mm^2^, after 150 mm 0.25 and 0.36 mm^2^, after 200 mm 0.04 and 0.36 mm^2^*). Fabrication of the thin film amorphous silicon sensors on the BF33-stack is described in detail in the next paragraph. After the realization of the sensors, the fluidic accesses to the microfluidic channel network are realized by means of laser drilling. At the bottom of the powderblasted pits cylindrical-shaped openings—diameter ca. 250 µm, height 200 ± 25 µm—are created with a laser (LaserTec, Barendrecht, The Netherlands). Finally, the stacks are diced into individual chips, using a protocol that ensures that no dicing debris/water could enter and/or pollute the fluidic network.

**Figure 2:**
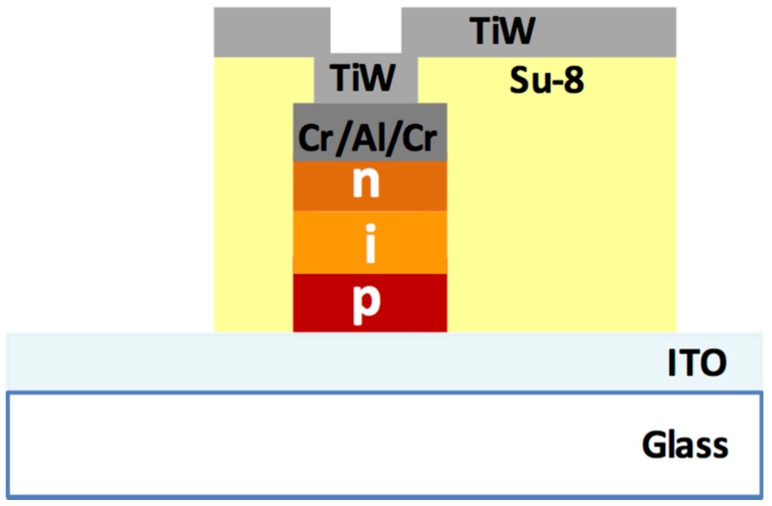
Cross section of the a-Si:H photosensor. Light is impinging through the glass substrate and the ITO (Indium Tin Oxide) bottom transparent electrode, before being absorbed by the p-i-n structure.

The a-Si:H photodiodes are fabricated by using standard microelectronic technologies. The photodiodes are p-type doped/intrinsic/n-type doped stacked structures grown by Plasma Enhanced Chemical Vapor Deposition (PECVD) at temperatures below 250 °C [32,33]. A cross section of the deposited photodiodes is shown in Figure 2.

The following steps are performed on the glass stack containing the microfluidic network:

1. cleaning of the outside of the stack by Piranha solution (H_2_SO_4_:H_2_O_2_ 3:1) and ultrasonic treatment in deionized water;
2. deposition (by magnetron sputtering) of a 100 nm-thick layer of Indium Tin Oxide (ITO) and subsequent patterning (by UV-photolithography). This transparent conductive oxide (TCO) is the front contact of the photodiode;
3. PECVD-deposition of the a-Si:H layers;
4. deposition (by evaporation) of a Cr/Al/Cr stacked layer, acting as bottom contact of the photosensor;
5. patterning of the Cr/Al/Cr stack and a-Si:H layers by means of wet and dry etching, respectively. This step defines the geometry of the photodiodes (which are aligned with respect to the microfluidic channels);
6. deposition and patterning of a 5 μm-thick layer of SU-8 3005 (MicroChem, Westborough, MA, USA), which acts as passivation and insulation layer on the lateral walls of the diodes. This patterning step also defines the via holes on top of the diodes; deposition and patterning, by magnetron sputtering, of a 200 nm-thick TiW layer for contacting the bottom contact of the diodes through the via holes and for the electrical connections to external pads.

**Figure 3:**
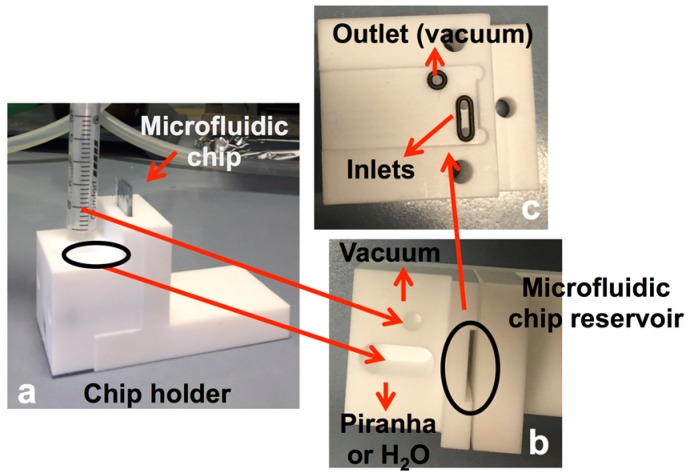
Pictures of the Teflon holder for Piranha activation of the channel interior: (**a**) chip holder overview, and top view of (**b**) bowl for inserting piranha or water, vacuum connection and reservoir for inserting the microfluidic chip and (**c**) chip holder reservoir for positioning chip inlets and outlet, respectively.

*Functionalization of the glass chips.* Chemical activation of the silanol groups on the inner walls of the microfluidic network was carried out with a Piranha solution that was flushed into the glass chips. In order to insert Piranha into the channel, the chip was positioned vertically in a home-made Teflon holder (Figure 3a). The bowl in correspondence of the inlets was filled with the piranha solution (Figure 3b,c). The Piranha solution was inserted in the channel by means of an underpressure by connecting the outlet of the chip to a vacuum pump (Figure 3b,c). The reduced pressure forced the Piranha to enter the channel network. After 20 min the bowl was rinsed with purified water, subsequently refilled with purified water and the outlet was reconnected to the vacuum pump to flush water through the chip to rinse the channel (20 min). The channel was then dried with a stream of nitrogen.

Poly(2-hydroxyethil methacrylate) (PHEMA) polymer brush layer on the inner wall of the microfluidic network was fabricated following previously reported procedures [13,34,35] (for details see supporting material section).

*Chemiluminescent reaction in the functionalized chip.* For all the experiments, the chips were positioned in a home-built fluidic holder designed for fitting fused silica capillaries (outer diameter 360 µm, inner diameter 250 µm) into the inlets and outlets of the chip by means of commercially available Upchurch Nanoport^TM^ assembly parts (Upchurch Scientific Inc., VWR International S.r.l., Milan, Italy). Sample solutions were flowed pressure-driven into the chip by means of a PHD 2000 series syringe pump (Harvard Apparatus, Crisel Instruments S.r.l, Rome, Italy) equipped with two syringes (250 μL flat tip; Sigma Aldrich, Milan, Italy). The syringes were connected to the fused silica capillaries by means of Nano-TightTM unions and fittings (Upchurch Scientific Inc., VWR International S.r.l., Milan, Italy).

The enzymatic reaction was conducted by means of the following solutions:

Syringe 1: mother solutions of luminol 0.1 M (0.06 g in 2.5 mL in phosphate buffer 10 mM pH = 7.4 and 0.5 mL NaoH 1 M) and 4-iodophenol (4-IOP) 0.1 M (0.066 g in 3 mL DMSO) were diluted in the same vial in phosphate buffer (10 mM pH = 7.4) to obtain the concentrations of 2 mM and 0.2 mM for luminol and 4-IOP, respectively.

Syringe 2: a mother solution of H_2_O_2_ 20 mM in phosphate buffer (10 mM pH = 7.4) was prepared from a commercially available H_2_O_2_ 30%. Subsequently, several solutions of H_2_O_2_ were prepared to have concentrations in the range of 75–700 μM.

The solutions were mixed into the microfluidic chip and the chemiluminescent reaction was followed/monitored by means of the on-chip photodiodes. In order to monitor the time evolution of the chemiluminescence-induced diode photocurrents, the photosensors were connected to a very low-noise electronic board [36] that is able to detect currents intensities in the pico-Ampere range. The connection between the photosensors and the electronic board was achieved through a card edge connector (SAMTEC MB1–150–01–L–S–02–SL), an electronic interface board, a substrate holder and a flex zip. In order to avoid electromagnetic interferences and influence of the ambient light on the readout, the electronics and the photosensor array are placed into a closed metallic box.

The nonlinear least-square fit of experimental data was carried out with the software SigmaPlot 12.0 (Systat Software, Inc., San Jose, CA, USA).

Limit of detection and quantifications (LOD and LOQ) were calculated (with a confidence interval 99.7% and >99.99%, respectively) using the following equations, 3 σ/m and 10 σ/m, where σ is the standard deviation of the blank and *m* is the slope of the calibration curve.

## 3. Results and Discussion

The main issue for the fabrication of this lab-on-chip is to harmonize the biochemical and microelectronic procedures with the fabrication of the microfluidic network, in order to achieve a final device that is sensitive enough for the analysis of low sample volumes.

In this work, we tailored and realized a glass chip in which the microfluidic network has been integrated with amorphous silicon photosensors to detect a biochemical reaction occurring through the chemical functionalization of the inner wall of the microchannel. In this manner, the fabrication of the device needs to accomplish the follow requirements:

- fabrication of the microfluidic network should not affect the photosensor performance;
- the photodiodes have to be optically aligned with the microfluidic network and their deposition should leave the channel clean for further utilization;
- the surface chemistry treatment should not affect the functionality of both the photodiodes and the channel network.

**Figure 4:**
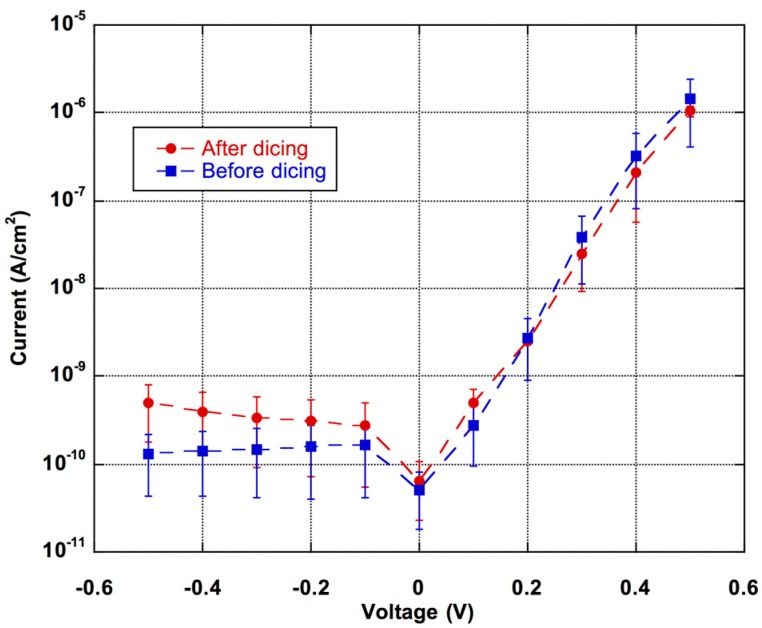
Current voltage characteristics of the a-Si:H photodiodes before (blue squares) and after (red circles) the dicing process. Symbols and bars refer, respectively, to the averaged values and to the standard deviations measured over five photodiodes.

*Photosensor Characterization*. After fabrication, the photodiodes are characterized through their current-voltage (I–V) curve in dark conditions, as well as their spectral response in the visible range. Before dicing the stack into individual chips, I–V characteristics are measured with a source measurement unit (SMU; Keithley, model 236) by applying a voltage and detecting the corresponding current. In order to avoid the effect of the defect trapping-detrapping mechanism, each voltage is applied for 5 s before performing current measurements. Each acquired current value is the average of 32 measurements, individually performed over a time interval of 50 ms. Results are reported as blue squares in Figure 4, where each point is the average current over 5 measurements performed on 5 different devices of the same batch. The error bars represent the standard deviations. As expected, an almost voltage-independent current for reverse bias conditions is observed and an exponential current increase in the forward region. Taking into account that photodiodes operate at small reverse bias (around 100 mV) the dark current gives a shot noise contribution of 1 fA/mm^2^.

Current-voltage characteristics are measured also after the dicing process and results reported as red circles in Figure 4. We observe a slight increase of the reverse current voltage, while the forward current does not show changes significantly. The increase of the dark current can be ascribed to mechanical stress induced in the amorphous silicon layers by the cutting process of the dicing and/or the laser drilling of the microfluidic accesses. However, the dark current noise increased to 1.2 fA/mm^2^ only.

The spectral response of the photodiodes is measured by using a double arm set-up. Radiation coming from a halogen lamp (ORIEL model 68735) is focused on the entrance pupil of a monochromator (Jobin-Yvon SPEX model 340E). The monochromator output is split in two beams: one beam is directed toward the chip to be characterized, the other on a calibrated crystalline photodiode (from Hamamatsu model 2550-2BNC). The current value achieved from the calibrated diode determines the number of photons (nph) effectively impinging on a-Si:H photodiodes during the measurements, thereby avoiding the effect of lamp intensity variations over time. Two source measurement units (Keithley, model 236) are used to measure the currents of the calibrated and on-chip diodes at 0 V applied bias. The current is acquired with the same settings as utilized for the I–V curves.

**Figure 5:**
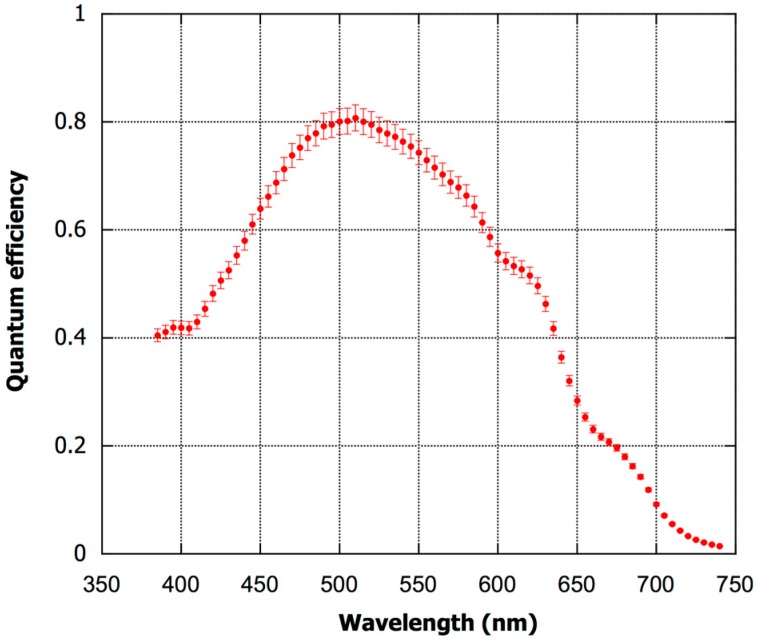
Spectral response of the photodiodes. Symbols and bars refer, respectively, to the averaged values and to the standard deviations measured over 5 photodiodes.

Results are given in Figure 5, which shows the quantum efficiency (QY) as a function of the wavelength. QY is defined as the ratio between the electrons collected at the a-Si:H diode electrodes and nph. The relation between QY and responsivity (R) is given in Equation (1):

R = λ*QY/1240(1)

where the wavelength λ is expressed in nm. By means of Equation (1) it can be calculated that in the full visible range the responsivity ranges between 100 and 330 mA/W. More specifically, at 425 nm, which is the wavelength at which the emission peak of the chemiluminescent reaction occurs, the responsivity is equal to 170 mA/W. The minimum detectable incident power, noise equivalent power (NEP), can be estimated from the following Equation (2):

NEP = I_Noise_/R_425_ (2)

where I_Noise_ is the total current noise (determined by the dark current and the electronics) and R_425_ the responsivity at 425 nm. Considering I_Noise_ to be ca. 4 fA, the minimum detectable power is around 0.02 pW.

In this work, as a proof of principle for on-chip detection with the properly working photodiodes, the lab-on-chip device was used as a system to detect hydrogen peroxide. For this purpose, the microfluidic channel is made functional by immobilization of HRP, which acts as an H_2_O_2_ biosensor. HRP is known to catalyze the reaction between luminol and hydrogen peroxide yielding a chemiluminescent signal with maximum emission at 425 nm.

*Microchannel Functionalization.* Once the fabrication of the device was completed, the channel was functionalized as follows: PHEMA was grown on the channel wall by atom transfer radical polymerization (ATRP), and the hydroxyl groups of PHEMA brush layer are reacted with succinic anhydride (SA), to obtain carboxylic functional moieties PHEMA-SA. Afterwards, a solution of n-hydroxysuccinimide (NHS) was flowed into the channel to achieve NHS esters functional groups (PHEMA-NHS). After rinsing with water, a solution of HRP was inserted into the channel and incubated over-night at 4 °C to form the PHEMA-HRP brush layer. The channel was rinsed with Blocking buffer for 30 min at the flow rate of 1 μL/min. The amount of immobilized HRP was evaluated using the bicinchoninic acid assay (BCA assay) and is found to be (11 ± 0.05) 10^−10^ μg/mm^2^. For further details, see the experimental procedure previously reported in reference [13].

**Figure 6:**
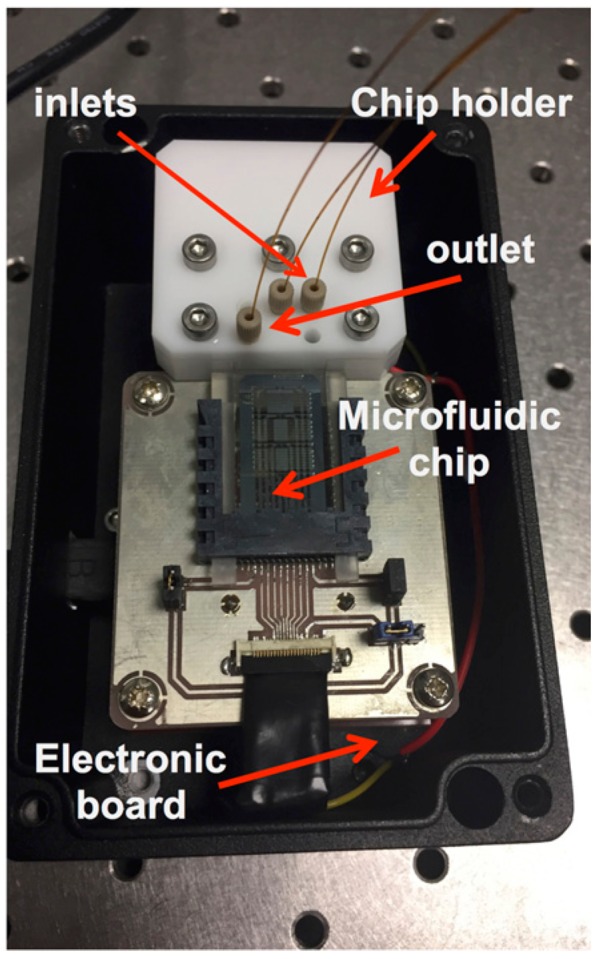
Picture of a horseradish peroxidase (HRP) functionalized chip connected to the electronic board for H_2_O_2_ detection.

*Enzymatic Reaction*. The functionalized chip is connected to the electronic board and covered with a metal lid (Figure 6). The chip has been tested for the detection of hydrogen peroxide using luminol and 4-IOP, which in the presence of HRP, acting as a catalyst, yields a chemiluminescent signal proportional to the concentration of H_2_O_2_. This chemiluminescent signal has been detected by the on-chip photosensors and quantified with a home-made program.

Indeed, in HRP-functionalized chips, the catalytic reaction occurs, as a CL-signal is clearly detected by the sensors. No signal is detected when the reagents are flowed through a chip with non-functionalized PHEMA brushes, proving that the enzyme is the catalyst. The functionalized chips could be used for one week (about 5 h per day) without any decrease of the enzymatic activity.

*Kinetic Analysis.* A set of kinetic experiments is carried out to investigate the chemiluminescence inside the microfluidic device. A pre-mixed solution of luminol (2.0 mM) and 4-IOP (0.20 mM), and a solution hydrogen peroxide (75–480 μM) were flowed into the channel via the two inlets into the functionalized chip. The solutions are mixed in the first part of the channel 50 mm before the position of the first photosensors (Figure 1). In order to carry out a quantitative kinetic analysis of the enzymatic reaction, the used concentration of luminol and 4-IOP are in excess compared to the concentration of hydrogen peroxide, which varied between 37.5 and 240 μM (after on-chip mixing). The experiments are conducted in continuous flow with rates ranging from 25 to 70 μL/min.

**Figure 7:**
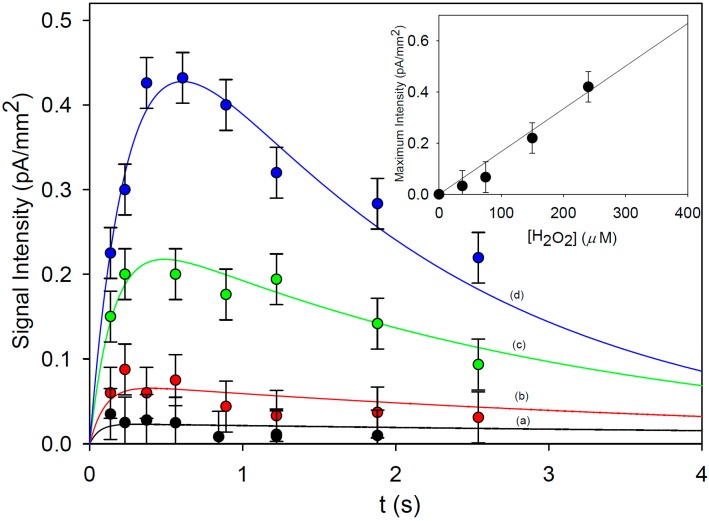
Signal intensity generated by the chemiluminescent decay of 2* versus residence time in the chip ([**1**] = 1.0 mM, [4-IOP] = 0.10 mM, T = 25 °C). The measurements were carried out at four different H_2_O_2_ concentrations: 37.5 μM (**a**), 75 μM (**b**), 150 μM (**c**), 240 μM (**d**). Data points are experimental, and the solid line is the plot of Equation (1) with best fit *k*_2_ = (2.5 ± 0.3) × 10^3^ M^−1^ s^−1^, *k*_c_ = 9.0 ± 3.2 s^−1^. Inset: plot of the maximum signal intensity versus the initial concentration of H_2_O_2_.

In Figure 7 the emission intensity detected by the sensors as a function of the residence time inside the chip is shown. All the photosensors were used for conducting the kinetic study. We observed that, for a given residence time, the value of chemiluminescence intensity is the same (within the experimental error) for the different sensors (see Table S1 in the supporting material section).

The emission intensity shows a fast increase in the early stage of the reaction, achieving a maximum, and then a slow decrease. This trend is observed for all the investigated concentrations of H_2_O_2_. The maximum of the luminescence intensity increases upon increasing the H_2_O_2_ concentration.

**Scheme 1:**
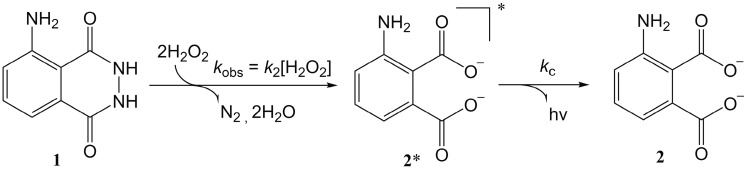
Mechanism for the chemiluminescent oxidation of luminol (**1**).

We can postulate that such experimental evidences are due to the fast chemiluminescent decay of the exited state of the 3-aminophtalate (2*), that is the intermediate of the luminol oxidation reaction (Scheme 1). If we assume the signal intensity to be proportional to the concentration of 2*, the standard integrated equations for two consecutive reactions [37,38] can be employed in the present case. The proper adaptation of these equations to the present reaction scheme affords Equation (3), which correlates the emission intensity *I* with the residence time *t*.

(3) $$I\left(t\right)=\frac{I_{o\;}k_{obs}}{k_{c}-k_{obs}}\left(e^{-k_{obs}t}-e^{-k_{c}t}\right)$$

In Equation (3) *k*_c_ and *k_obs_* = *k*_2_[H_2_O_2_] are the rate constants of the two reaction steps as defined in Scheme 1, and *I_o_* is a constant proportional to the initial luminol concentration.

A nonlinear least-squares multifit of experimental data in Figure 7 to Equation (3) gives the following values of best fit parameters: *k*_2_ = (2.5 ± 0.3) × 10^3^ M^−1^ s^−1^, *k*_c_ = 9.0 ± 3.2 s^−1^. The nice fit of data points to Equation (3) confirms the hypothesis that the observed experimental evidence is due to the reaction mechanism in Scheme 1. Furthermore, the plot of the maximum signal intensity detected by the sensors as a function of the initial concentration of H_2_O_2_ (Figure 7, inset) shows a linear trend (see also Figure S1 in supplementary material file).

The slope of the calibration curve is (1.67 ± 0.12) 10^−3^ pA mm^−2^ μM^−1^ and the limit of detection (LOD) and quantification (LOQ) for H_2_O_2_ are 18 and 60 μM, respectively. These values are comparable to the best performances reported in the literature for chemiluminescent-based optofluidic sensors [20,21,24]. 

Even though other authors using absorbance [39] or fluorescent [40] detection methods achieved better sensitivities in the detection of hydrogen peroxide, it is important to highlight the following features of our proposed system:

- the chemiluminescence approach, used in our work, does not need an excitation source, leading to a lower power consumption and a more compact system;
- the distance between the site where the chemiluminescence occurs and the detectors (the a-Si:H photosensors) is reduced to 700 microns (the thickness of the glass hosting the photosensors) which minimizes the optical loss to the light diffusion;
- the alignment between the radiation source (the inner of the channel) and the detectors is done automatically during the device fabrication;
- our system is completely re-usable (without any disposable part) just by cleaning with piranha the microfluidic channel. This is possible thanks to the use of the all-glass microfluidic approach. Therefore, such a cleaning process is not possible with a PDMS network.

## 4. Conclusions

In this work, we report on the development and the testing of an innovative lab-on-chip device for the monitoring of chemiluminescent reactions. Various aspects are combined in these chips, such as a microfluidic channel, a chemical functionalization procedure of the channel inner wall with polymer brushes required for the immobilization of HRP enzyme and on-chip a-Si:H photosensors for the detection of the chemiluminescent signals.

The fabricated devices are tested for the real-time monitoring of the CL-signal derived from the reaction of luminol, 4-iodophenol and hydrogen peroxide. The obtained results show that the chemiluminescent signal is proportional to the concentration of H_2_O_2_. The kinetic study demonstrates the operation of a two-step reaction: (i) the formation of the intermediate 3-aminophtalate in its exited state and (ii) the conversion of the same molecule into the ground state. A linear calibration curve for H_2_O_2_ was observed, whose elaboration provide LOD and LOQ values of 18 and 60 µM, respectively.

This lab-on-chip may have several applications for biochemical analysis: antibodies labeled with HRP are widely used for ELISA assays and they can be employed to detect different targets possibly immobilized directly [11] or indirectly (thorough another antibody) to the channel interior. Moreover, the co-immobilization of HRP with oxidative enzymes might allow the on-chip detection of several analytes whose quantification is important for health-care and diagnostics. Finally, upon the use of capillary force instead of pressure-driven flow, we envision its utilization as a portable point of care system (POC), which is the subject of our current research.

## Supplementary Materials

The following are available online at www.mdpi.com/2079-6374/7/4/58/s1, Figure S1: Calibration curve extended to higher H_2_O_2_ concentrations showing a saturation profile at concentrations higher than 250 µM, Table S1: Data of the kinetic plot of Figure 7.
