# Supplementary material for: An All-Glass Microfluidic Network with Integrated Amorphous Silicon Photosensors for on-Chip Monitoring of Enzymatic Biochemical Assay
Source: Biosensors (Basel). 2017 Dec 5;7(4):58. doi: 10.3390/bios7040058 (PMC5746781; doi:10.3390/bios7040058)
Supplement: Supplementary file 1 [file biosensors-07-00058-s001.pdf]

biosensors

Supplementary Material

An All-Glass Microfluidic Network with Integrated Amorphous Silicon Photosensors for on-Chip Monitoring of Enzymatic Biochemical Assay

Francesca Costantini [1,2,*], Roald M. Tiggelaar [3,4], Riccardo Salvio [2], Marco Nardecchia [1], Stefan Schlautmann [3], Cesare Manetti [5], Han J.G.E. Gardeniers [3], Giampiero deCesare [6], Domenico Caputo [6] and Augusto Nascetti [1]

1 School of Aerospace Engineering, Sapienza University of Rome, via Salaria n. 851/881, 00138 Rome, Italy; marco.nardecchia@uniroma1.it (M.N.), augusto.nascetti@uniroma1.it (A.N.)
2 Department of Chemistry, Sapienza University of Rome, p.le Aldo Moro n.5, 00185 Rome, Italy; riccardo.salvio@uniroma1.it
3 Mesoscale Chemical Systems, MESA+ Institute for Nanotechnology, University of Twente, P.O. Box 217, 7500 AE Enschede, The Netherlands; r.m.tiggelaar@utwente.nl (R.M.T.); s.schlautmann@utwente.nl (S.S.); j.g.e.gardeniers@utwente.nl (H.J.G.E.)
4 NanoLab cleanroom, MESA+ Institute for Nanotechnology, University of Twente, P.O. Box 217, 7500 AE Enschede, The Netherlands
5 Department of Environmental Biology, Sapienza University of Rome, p.le Aldo Moro n.5, 00185 Rome Italy; cesare.manetti@uniroma1.it
6 Department of Information Engineering, Electronics and Telecommunications, Sapienza University of Rome, Via Eudossiana, 18, 00184 Rome, Italy; giampiero.decesare@uniroma1.it (G.d.C.); domenico.caputo@uniroma1.it (D.C.)
* Correspondence: Francesca.costantini@uniroma1.it; Tel.: +39-064-991-3576

1. Materials

All reagents were purchased from Aldrich Chemicals. 2-Hydroxyethyl methacrylate (HEMA) was distilled prior to use, whereas the other chemicals were used without further purification. 2-Bromo-2-methyl-propionic acid 3-trichlorosylanyl-propyl ester was synthesized following a reported procedure [1,2]. Ethanol (analytical reagent grade) was used without further purification, while toluene was distilled over sodium. Water was purified with a Milli-Q pulse (MILLIPORE, R 1⁄4 18.2 MU cm) ultra-pure water system.

2. Functionalization of the Microfluidic Channel

Afterwards, a solution of 0.2% 2-bromo-2-methyl-propionic acid 3-trichlorosylanyl-propyl ester in toluene was flowed through the channel (one night; 0.1 mL/min) to form a self assembled monolayer [3]. Subsequently, the channel was rinsed with toluene and dried with a stream of nitrogen. A solution of 10 mL 2-hydroxyethyl methacrylate (HEMA) and 10 mL of MilliQ water was degassed by bubbling with dry nitrogen (N_2) for 30 min and transferred in a schlenk tube in which it was stored under an argon environment. Copper(I) chloride (0.055 g), copper(II) bromide (0.036 g) and 2.2′-dipyridyl (0.244 g) were added. To dissolve the solid, the mixture was stirred for 10 min (while degassing), which yielded a dark brown solution. The solution was sonicated till complete dissolution of the solid, then sucked into a Hamilton syringe and inserted in the microfluidic chip in which it was kept inside under no flow conditions (for 60 min) in order to form the PHEMA brush film. The channel was rinsed with ethanol and water (10 min; 1 μL/min) to completely remove the copper residues, and dried with a nitrogen flux. A tetrahydrofurane (THF) solution (2 mL) of succinic anhydride (100 mg) and triethylamine (100 mL) was flowed through the chip (24 h; 0.1 μL /min), followed by rinsing with ethanol (10 min; 1 μL /min). After this step, a water solution

(1 mL) of N-hydroxysuccinimide (NHS) (15 mg) and 1-ethyl-3-(3-dimethylaminopropyl) carbodiimide (EDC) (75 mg) was flowed in the channel (0.1 μL/min; 1 h), followed by rinsing with purified water. After drying the channel with a stream of nitrogen, a phosphate buffer solution of 10 mM and pH=7.5 (this buffer was prepared by diluting a 25 mM phosphate buffer solution pH 7.5, NaH_2PO_4 5 mM; Na_2HPO_4 20 mM) containing HRP (0.5 mg/mL) was flushed through the chip and kept inside for one night at 4 °C in order to immobilize HRP onto the activated PHEMA-brushes formed onto the channel. Finally, the channel was rinsed with Blocking buffer (Tris 10 mM pH=8) for 30 min (1 μL/min), in order to remove all enzyme molecules that did not link covalently to the polymer film and to block the non-reacted NHS-ester moieties.

Table S1. Data of the kinetic plot of Figure 7. In the table are reported also the flow rates and the sensors used for the measurements. The intensity values reported come from the average of the intensities of the indicated sensors normalized for the sensor area (error based on standard deviation of three measurements using different devices is ca. 10% for all the values).

**[H_2O_2] μM**	**Flow Rate (μL/min)**	**Sensors**	**Time (s)**	**Intensity (pA/mm^2)**
37.5	70	1-2	0.1357	0.035
	50	1-2	0.23	0.025
	25	1-2	0.56	0.028
	70	5-6	0.6071	0.0248
	50	5-6	0.89	8.33E-03
	25	5-6	1.88	9.78E-03
75	70	1-2	0.1357	0.06
	50	1-2	0.23	0.0875
	70	3-4	0.3714	0.06
	50	3-4	0.56	0.075
	50	5-6	0.89	0.044
	50	7-8	1.22	0.033
	25	5-6	1.88	0.037
	25	7-8	2.54	0.0311
150	70	1-2	0.1357	0.15
	50	1-2	0.23	0.2
	50	3-4	0.56	0.2
	50	5-6	0.89	0.176
	50	7-8	1.22	0.194
	25	5-6	1.88	0.1417
	25	7-8	2.54	0.0933
225	70	1-2	0.1357	0.225
	50	1-2	0.23	0.3
	70	3-4	0.3714	0.426
	70	5-6	0.6071	0.432
	50	5-6	0.89	0.4
	50	7-8	1.22	0.32
	25	5-6	1.88	0.2833
	25	7-8	2.54	0.2194

350	70	1-2	0.1357	0.2315
	50	1-2	0.23	0.3512
	70	3-4	0.3714	0.4418
	70	5-6	0.6071	0.4832
	50	5-6	0.89	0.4356
	50	7-8	1.22	0.3941
	25	5-6	1.88	0.2946
	25	7-8	2.54	0.2153

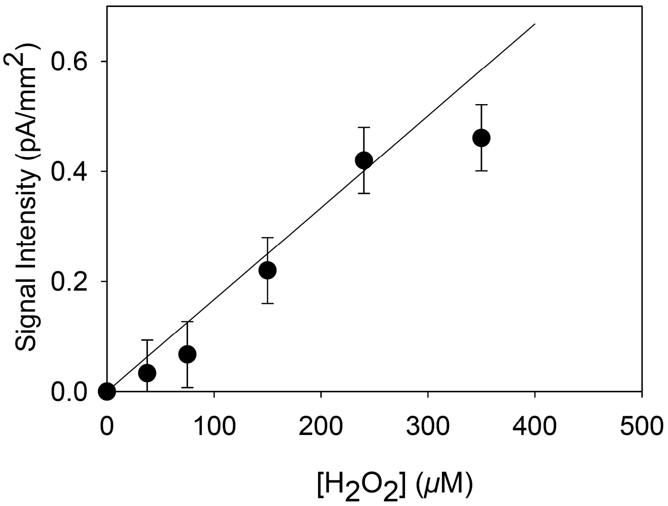

Figure S1. Calibration curve extended to higher H_2O_2 concentrations showing a saturation profile at concentrations higher than 250 μM. This trend is probably due to the limited amount of enzyme immobilized in the surface of the microchannel network.

References

1. Costantini, F.; Bula, W.P.; Salvio, R.; Huskens, J.; Gardeniers, H.; Reinhoudt, D.N.; Verboom, W. Nanostructure based on polymer brushes for efficient heterogeneous catalysis in microreactors. J. Am. Chem. Soc. 2009, 131, 1650-1651.
2. Savelli, C.; Salvio, R. Guanidine-based polymer brushes grafted onto silica nanoparticles as efficient artificial phosphodiesterases. Chem. Eur. J. 2015, 21, 5856-5863.
3. Fasolato, C.; Domenici, F.; Sennato, S.; Mura, F.; De Angelis, L.; Luongo, F.; Costantini, F.; Bordi, F.; Postorino, P. Dimensional scale effects on surface enhanced raman scattering efficiency of self-assembled silver nanoparticle clusters. Appl. Phys. Lett. 2014, 105.
